# Supplementary material for: Ultrastructural Characterization of Developmental Stages and Head Sensilla in Portici okadai, Vector of Thelazia callipaeda
Source: Insects. 2025 May 20;16(5):539. doi: 10.3390/insects16050539 (PMC12112477; doi:10.3390/insects16050539)
Supplement: Supplementary file 1 [file insects-16-00539-s001.zip › insects-3610295-supplementary.pdf]

Table S1. Abundance and distribution of sensilla on the antennae and maxillary palps of *P. okadai*

Tissue	Type of Sensilla	Subtype	Male	Female
Antennae	Chaetica sensilla	Ch Ⅰ	1.0±0.0	1.3±0.3
		ChⅡ	3.3±0.7	4.0±0.6
	Trichod sensilla	T	11.3±0.9	12.7±1.5
	Intermediate sensilla	I	9.3±0.7	10.3±0.9
	Coeloconic sensilla	C	2.7±0.3	3.0±0.6
	Basiconic sensilla	TB	1.7±0.3	2.0±0.6
		LB	3.3±0.7	4.0±0.6
Maxillary palp	Chaetica sensilla	Ch Ⅰ	1.3±0.3	1.3±0.3
		ChⅡ	2.3±0.3	2.0±0.6
	Basiconic sensilla	TB	8.3±0.3	8.0±0.6
		LB	9.7±0.3	10.0±0.6
		SB	7.3±0.9	8.3±0.3

Data are the mean ± S.E. For the number of different types of sensilla on antenna and maxillary palpus of *P. okadai*; the values of Ch Ⅰ, ChⅡ, T, I, C, TB, LB, and SB are mean density number per unit area of 100 μm^2 on the surface of the antenna and maxillary palp. Ch, chaetica sensilla; T, trichoid sensilla; I, Intermediate sensilla; C, Coeloconic sensilla; TB, LB, and SB, basiconic sensilla. No statistically significant male-female comparisons were made at the same site (independent-samples *t*-test; $p = 0.05$).
